# Germacrone Attenuates Hepatic Stellate Cells Activation and Liver Fibrosis via Regulating Multiple Signaling Pathways

**DOI:** 10.3389/fphar.2021.745561

**Published:** 2021-10-05

**Authors:** Zhiyong Li, Zhilei Wang, Fang Dong, Wei Shi, Wenzhang Dai, Jing Zhao, Qiang Li, Zhi-e Fang, Lutong Ren, Tingting Liu, Ziying Wei, Wenqing Mou, Li Lin, Yan Yang, Xiaohe Xiao, Li Ma, Zhaofang Bai

**Affiliations:** ^1^ School of Pharmacy, Chengdu University of Traditional Chinese Medicine, Chengdu, China; ^2^ Department of Hepatology, Fifth Medical Center of Chinese PLA General Hospital, Beijing, China; ^3^ TCM Regulating Metabolic Diseases Key Laboratory of Sichuan Province, Hospital of Chengdu University of Traditional Chinese Medicine, Chengdu, China; ^4^ School of Public Health and Health Management, Shandong First Medical University and Shandong Academy of Medical Sciences, Shandong, China; ^5^ China Military Institute of Chinese Materia, Fifth Medical Center of Chinese PLA General Hospital, Beijing, China; ^6^ School of Traditional Chinese Medicine, Capital Medical University, Beijing, China

**Keywords:** ROS: reactive oxygen species, HSCs: hepatic stellate cells, germacrone, TGF-β/Smad pathway, apoptosis

## Abstract

Liver fibrosis is an abnormal proliferation of connective tissue in the liver caused by various pathogenic factors. Chronic liver injury leads to release of inflammatory cytokines and reactive oxygen species (ROS) from damaged hepatocytes, which activates hepatic stellate cells (HSCs) to secrete extracellular matrix proteins, thereby leading to fibrosis. Thus, inhibition of hepatocyte injury and HSC activation, and promotion of apoptosis of activated HSCs are important strategies for prevention of liver fibrosis. In this study, we showed that the germacrone (GER), the main component in the volatile oil of zedoary turmeric, inhibited hepatic fibrosis by regulating multiple signaling pathways. First, GER improved the cell survival rate by inhibiting the production of ROS after hepatocyte injury caused by acetaminophen (APAP). In addition, GER inhibited the activation of HSCs and expression of collagen I by blocking TGF-β/Smad pathway in LX-2 cells. However, when the concentration of GER was higher than 60 μM, it specifically induced HSCs apoptosis by promoting the expression and activation of apoptosis-related proteins, but it had no effect on hepatocytes. Importantly, GER significantly attenuated the methionine- and choline-deficient (MCD) diet-induced liver fibrosis by inhibiting liver injury and the activation of HSCs *in vivo*. In summary, GER can not only protect hepatocytes by reducing ROS release to avoid the liver injury-induced HSC activation, but also directly inhibit the activation and survival of HSCs by regulating TGF-β/Smad and apoptosis pathways. These results demonstrate that GER can be used as a potential therapeutic drug for the treatment of liver fibrosis.

## Introduction

In recent years, liver fibrosis has become a hot spot in liver disease research, and the complex mechanism of liver fibrosis formation has been revealed from many aspects (Parola and Pinzani, 2019). In the process of liver fibrosis, the dead and dying parenchymal cells release the danger signals, including inflammatory factors, damage associated molecular patterns (DAMPs), and reactive oxygen species (ROS), which can contribute to the activation of the hepatic stellate cells (HSCs) (Pellicoro et al., 2014). The ROS-generating enzymes, NADPH oxidase 1 (NOX1), NOX2, or NOX4, can induce liver fibrosis by activating HSCs (Lan et al., 2015). Importantly, HSCs are the most direct and relevant cell type for the formation of liver fibrosis. They are nonparenchymal cells of the liver, located in the space between liver sinusoidal endothelial cells (LSECs) and hepatocytes, and account for about 10% of the intrinsic cells of the liver (Higashi et al., 2017). Under the physiological conditions, the main function of HSCs is to metabolize and store retinol in lipid droplets in the cytoplasm; HSCs exhibit a nonproliferating quiescent phenotype, quite different from other cell morphologies. When the liver is damaged, the quiescent HSCs are activated and they differentiate into myofibroblasts, which secrete collagen and other components of the extracellular matrix (ECM). This transformation is an important step in the occurrence of liver fibrosis (Tsuchida and Friedman, 2017). The activated HSCs transform the main components of the ECM from type IV collagen, heparan sulfate proteoglycan (HSPG), and laminin (LN) to type I and III collagen, resulting in an increase in the density and hardness of the ECM (Cordero-Espinoza and Huch, 2018).

The activation of HSCs is related to the cytokines such as connective tissue growth factor (CTGF), transforming growth factor (TGF), platelet-derived growth factor (PDGF) and vascular endothelial growth factor (VEGF) released by liver parenchymal cells (Yang et al., 2014; Dewidar et al., 2019). TGF-β/Smad signaling is known as the classic HSC activation pathway. Under the stimulation by TGF-β1, the principal transforming growth factor isoform, the overexpressed TGF-β1 binds to HSC surface receptors and mediates the activation of HSCs. Then, the Smad2 and Smad3 are recruited to the TGF-β receptor and finally activated through phosphorylation. The activated Smad complex simultaneously recruits transcription co-activation molecules (such as P300/CBP and MSG1) and co-inhibitory molecules (TGIF and Ski/Sno N). Subsequently, phosphorylated Smad2/3 and Smad4 form a complex and then translocate into the nucleus to regulate the transcription of downstream pro-fibrosis genes, leading to liver fibrosis (Greenwel et al., 1997; Ghosh et al., 2000; Hata and Chen, 2016; Yoshida et al., 2018). In contrast, in the process of feedback regulation, Smad7 acts as a negative regulator (Bian et al., 2014). In addition, TGF-β1 can also activate the mitogen-activated protein kinase (MAPK) signaling pathway to promote HSC activation. TGF-β1 can regulate the activated MAPK signaling pathway, including extracellular signal-regulated kinase (ERK), P38 MAPK, and c-Jun N-terminal kinase (JNK), thereby promoting HSC activation (Engel et al., 1999; Hanafusa et al., 1999; Hernandez-Aquino and Muriel, 2018). Therefore, blocking HSC activation and promoting HSC apoptosis may be the strategies to prevent liver fibrosis.

The Liuweiwuling tablet, a traditional Chinese medicine preparation, has a significant effect on the treatment of liver fibrosis (Liu et al., 2018). Zedoary turmeric oil, the main medicinal material of Liuweiwuling prescriptions, has a significant antifibrotic effect (Jiang et al., 2005). In this study, we found that germarcone (GER), one of the main components of the volatile oil of zedoary turmeric, inhibited hepatic fibrosis by regulating multiple signaling pathways. We demonstrated that GER ameliorates liver fibrosis by inhibit the survival and activation of HSCs through apoptosis and TGF-β/Smad pathway *in vitro*. We further found that GER is able to reduce the release of ROS caused by APAP in L02, which plays an important role in protecting hepatocytes. Finally, GER also improves liver injury and prevents liver fibrosis through TGF-β/Smad pathway caused by methionine- and choline-deficient (MCD) diet.

## Materials and Methods

### Reagents and Antibodies

Reagents: germacrone (TOPSCIENCE, T2945), acetaminophen (APAP, MCE, HY-66005), N-acetyl-L-cysteine (NAC, MCE, HY-B0215), hydrogen peroxide solution (H_2_O_2_, Sigma, 323381), Dulbecco’s modified Eagle medium (DMEM, Macgene, CM10013), fetal bovine serum (FBS, Biological Industries, 04-001-1ACS), 1% penicillin–streptomycin (Macgene, CC004), 0.25% trypsin-EDTA (Macgene, CC012.100), 0.05% trypsin-EDTA (Macgene, C01712), TGF-β1 (PeproTech, AF-100-21C), Cell Counting Kit-8 (CCK-8, Dojindo, CK04), PE Annexin V Apoptosis Detection Kit I (BD Pharmingen^™^, 559763), MitoSOX^™^ red mitochondrial superoxide indicator (Invitrogen^™^, M36008), Carboxymethyl Cellulose-Na(CMC-Na, SCR, 9004-32-4), and methionine- and choline-sufficient, methionine- and choline-deficient (MCS/MCD, Dyets, MCDAA).

Antibodies: COL1A1 (R&D, AF6220), α-SMA (CST, 19245), P-Smad3 (CST, 9520), Smad3 (CST, 9523), PARP (CST, 9542S), cleaved PARP (CST, 5625S), caspase-3 (CST, 9665S), cleaved caspase-3 (CST, 96615), Bcl-2 (Abcam, ab32124), Bax (Abcam, ab32503), and GAPDH (GeneTex, GTX100118).

### Cell Culture and Treatment

LX-2 human HSCs were purchased from Shanghai YuBo Biotechnology Co., Ltd. (YB-H3614), whereas human hepatocyte LO2 cells were kindly provided by Dr. Tao Li from National Center of Biomedical Analysis (Beijing, China). All the cells were cultured in 5% CO_2_ incubator at 37°C using DMEM containing 10% FBS and 1% penicillin–streptomycin.

Cell viability test: LX-2 at 2.5 × 10^4^ cells/well and LO2 at 2 × 10^4^ cells/well were seeded in 96-well plates overnight. Then, the cells were incubated with different concentrations of GER (0–200 μM) for 24 h, and the cell viability was detected by CCK-8.

Drug effect detect: LX-2 at 7.5 × 10^4^ cells/well were seeded in 24-well plates overnight. The LX-2 cells were starved with serum-free medium for 18 h. Then, the cells were pretreated with 10, 20, and 40 μM GER for 1 h. After stimulation with TGF-β1 (10 ng/ml) for 24 h, the cell lysate was collected for western blot and RT-qPCR analysis.

LX-2 cells at 7.5 × 10^4^ cells/well were cultured in 24-well plates overnight. The cells were treated with 40–120 μM GER for 24 h, and the cell lysate was collected for western blot analysis. Alternatively, after the same treatment, the cells were collected and stained in line with the PE Annexin V Apoptosis Detection Kit I instructions, and the apoptotic cells were detected by flow cytometry.

LO2 cells at 6 × 10^4^ cells/well were seeded in a 24-well plate overnight. The cells were pretreated with 10, 20, and 40 μM GER for 12 h, and then incubated with 20 mM APAP for 12 h. All the cells were collected and stained with MitoSOX red mitochondrial superoxide indicator. Finally, the mitochondrial ROS was detected by flow cytometry.

### Mouse Model of Liver Fibrosis

Male C57BL/6 mice (6–7 weeks old) were purchased from SPF Biotechnology Co., Ltd. (Beijing, China). They were placed in the animal experiment center of the Fifth Medical Center of Chinese PLA General Hospital, Beijing 100039, China. This study was reviewed and approved by the animal ethics committee of the Fifth Medical Centre, Chinese PLA General Hospital (Beijing, China). All animals were fed adaptively under controlled temperature (25 ± 3 °C) and 12-h dark/light cycle, during which sufficient food and water were provided. GER for the *in vivo* study was prepared with PBS containing 1% DMSO and 0.5% CMC-Na.

The animal model of liver fibrosis was developed using the MCS/MCD diet. All the mice were divided into five groups (*n* = 8). The control group was fed MCS diet and four experimental groups were fed MCD diet. Two weeks before the development of the model, all of the groups were fed MCS diet for transition, and then the experimental group gradually increased MCD diet. Two weeks later, the experimental groups were fed only MCD diet and began to receive drug treatment at the same time. The control group and the model group were given vehicle solvent, the positive drug group was given 0.2 mg/ml colchicine (COL), and the two treatment groups were given 25 or 50 mg/kg GER. The dosage of administration was 0.2 ml for per mouse through intragastric gavage (ig) every day for 6 weeks, and they were weighed twice a week. After the last administration, the mice fasted for 12 h and were killed by neck dislocation. The serum and liver were collected for detection and analysis.

### Liver Function Parameter Tests

The levels of aspartate aminotransferase (AST) and alanine aminotransferase (ALT) in serum were detected in line with the instructions of AST (Nanjing JianChen Bioengineering Institute, C010-2-1) and ALT (Nanjing JianChen Bioengineering Institute, C009-2-1) kits.

### Liver Fibrosis Parameter Tests

The levels of hydroxyproline (Hyp) and PIIINP (procollagen type III N-terminal propeptide) were detected in line with instructions of ELISA Kit for hydroxyproline (Cloud-Clone Corp, CEA621Ge) and mouse procollagen type III N-terminal propeptide ELISA Kit (Novus Biologicals, NBP2-81204).

### Histological and Immunohistochemical Assays

The liver tissue was fixed with 10% formalin solution, embedded in paraffin, cut into 7-mm-thick sections, and stained with hematoxylin-eosin (HE) and Masson. For immunohistochemical staining, the paraffin-embedded sections were incubated with the primary antibodies α-SMA and COL1A1, followed by incubation with the HRP-linked secondary antibodies. Finally, the quantitative analysis of α-SMA and COL1A1 were performed using K-Viewer software (K-Viewer V1, 1.5.3.1, KFBIO).

### Western Blot Analysis

The collected total cellular proteins were separated by 10% SDS-PAGE gel electrophoresis and transferred to the polyvinylidene fluoride (PVDF) membrane, which was blocked with 5% skimmed milk for 1 h. Then, the membrane was incubated with the following antibodies: COL1A1, α-SMA, P-Smad3, Smad3, caspase-3, cleaved caspase-3, PARP, cleaved PARP, Bcl-2, and Bax. The GAPDH was used as a loading control. After incubating the linked-HRP secondary antibody, the bands were visualized using an ECL detection reagent and then develop it with the film.

We added 50 mg of liver tissue to 1 ml of RIPA lysate (including protease inhibitor) and homogenized at the frequency of 100 Hz for 90 s. The tissue lysate was placed on ice for 30 min and the supernatant was absorbed. The protein level was quantified by the cellular western blot method, and the following antibodies were incubated: α-SMA, Smad3, and P-Smad3. The β-actin was used as the loading control. After incubating the linked-HRP secondary antibody, the bands were visualized using an ECL detection reagent and then develop it with the film.

### Quantitative Real-Time Polymerase Chain Reaction

TRlzol (Sigma, 93289) was used to extract cell total RNA or tissue total RNA in line with the manufacturer’s protocol. Then, we reversed the extracted RNA to cDNA by RT Master Mix for qPCR (MCE, HY-K0510). Real-time PCR was performed with an Applied Biosystems ViiA6 Real-time PCR system using the SYBR Green qPCR Master Mix (MCE, HY-K0501). The PCR primer sequences are shown in Tables 1, 2. The expression of related genes was calculated by ΔΔCt relative to GAPDH.

**TABLE 1:** Human primers for quantitative real-time PCR analysis.

Name		Sequence (5′ 3′)
ACTA2	Forward primer	CAG​GGC​TGT​TTT​CCC​ATC​CAT
	Reverse primer	GCC​ATG​TTC​TAT​CGG​GTA​CTT​C
COL1A1	Forward primer	GTC​GAG​GGC​CAA​GAC​GAA​G
	Reverse primer	CAG​ATC​ACG​TCA​TCG​CAC​AAC
Smad3	Forward primer	CCA​TCT​CCT​ACT​ACG​AGC​TGA​A
	Reverse primer	CAC​TGC​TGC​ATT​CCT​GTT​GAC
MMP-2	Forward primer	GTG​AAG​TAT​GGG​AAC​GCC​G
	Reverse primer	GCC​GTA​CTT​GCC​ATC​CTT​CT
GAPDH	Forward primer	AGC​CAC​ATC​GCT​CAG​ACA​C
	Reverse primer	GCC​CAA​TAC​GAC​CAA​ATC​C

**TABLE 2:** Mouse primers for quantitative real-time PCR analysis.

Name		Sequence (5′ 3′)
ACTA2	Forward primer	TCAGCGCCTCCAGTTCCT
	Reverse primer	AAA​AAA​AAC​CAC​GAG​TAA​CAA​ATC​AA
COL1A1	Forward primer	ACG​TCC​TGG​TGA​AGT​TGG​TC
	Reverse primer	CAG​GGA​AGC​CTC​TTT​CTC​CT
Smad3	Forward primer	AGG​GGC​TCC​CTC​ACG​TTA​TC
	Reverse primer	CAT​GGC​CCG​TAA​TTC​ATG​GTG
TGF-β1	Forward primer	AGA​CCA​CAT​CAG​CAT​TGA​GTG
	Reverse primer	GGT​GGC​AAC​GAA​TGT​AGC​TGT
GAPDH	Forward primer	AAT​GGA​TTT​GGA​CGC​ATT​GGT
	Reverse primer	TTT​GCA​CTG​GTA​CGT​GTT​GAT

### Data and Statistical Analysis

The data were presented as the mean ± standard error of mean (SEM). GraphPad Prism 5.0 (GraphPad Software, San Diego RRID:SCR_002798) was used for statistical analysis. A two-tailed unpaired Student’s *t* test for two groups or one-way ANOVA for multiple groups was conducted to evaluate the significant differences. A *p*-value < 0.05 was considered statistically significant.

## Results

### Screening of Effective Components Inhibiting HSC Activity in Zedoary Turmeric Oil

**FIGURE 1:**
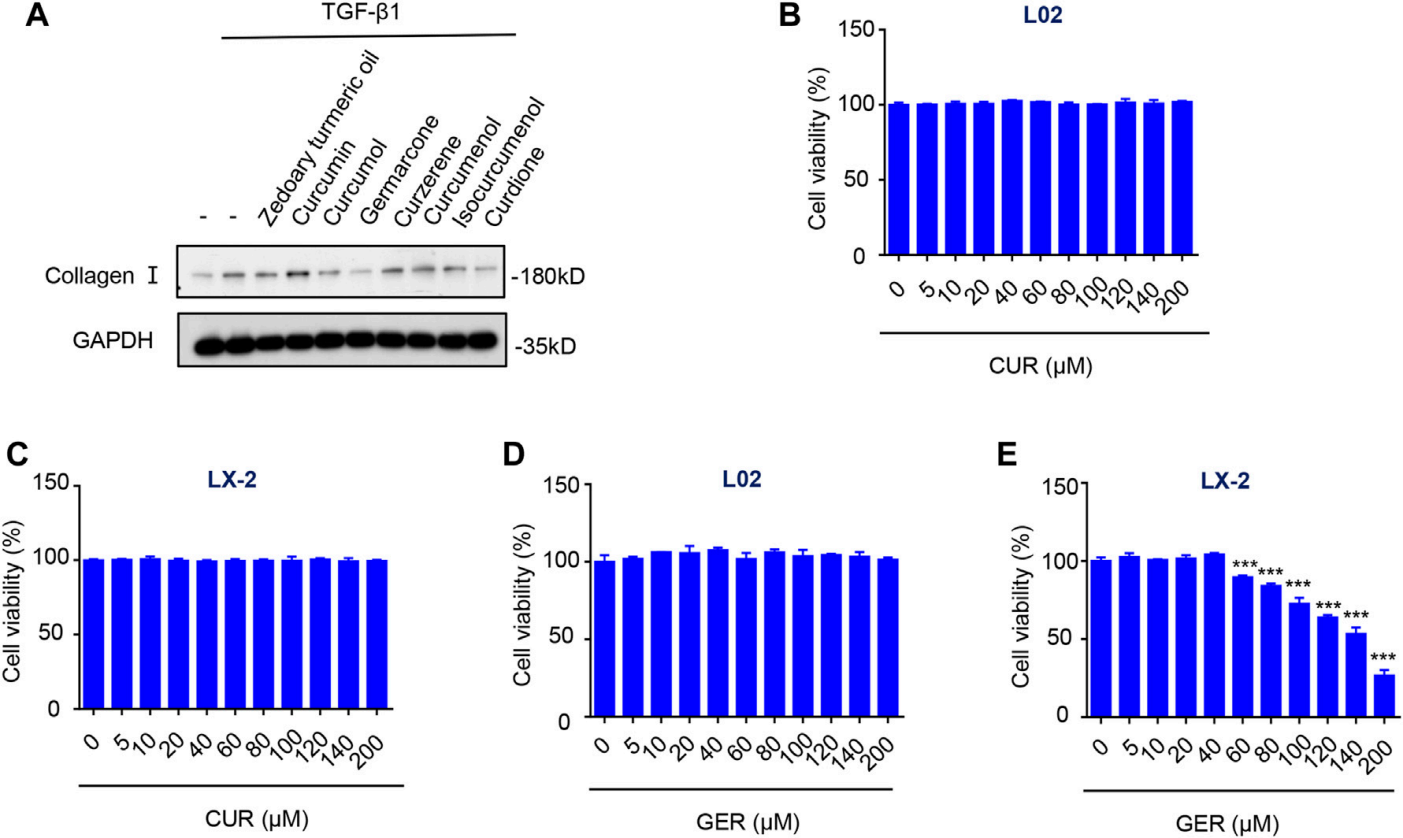
Screening the effective components in zedoary turmeric oil which inhibit the activity of HSCs. **(A)** LX-2 cells were treated with Zedoary turmeric oil (0.2 μL/ml) and Curcumin, Curcumol, Germarcone (GER), Curzerene, Curcumenol, Isocurcumenol, and Curdione (CUR) (40 μM) for 25 h, and then were stimulated by TGF- β (10 ng/ml) for 24 h. The expression of collagen I was detected by western blot, and GAPDH was used as the loading control. **(B)** L02 cells were treated with CUR for 24 h, then the cell viability was detected by CCK-8 (*n* = 3). **(C)** LX-2 cells were treated with CUR for 24 h, then the cell viability was detected by CCK-8 (*n* = 3). **(D)** L02 cells were treated with GER for 24 h, then the cell viability was detected by CCK-8 (*n* = 3). **(E)** LX-2 cells were treated with GER for 24 h, then the cell viability was detected by CCK-8 (*n* = 3). All the results were compared with control group. All data are presented as means ± SEM. ***p < 0.001 vs. the control group.

We found that the Liuweiwuling tablet, a prescription of traditional Chinese medicine, inhibited hepatic fibrosis in rats treated with carbon tetrachloride, and it was reported that zedoary turmeric oil was the main antifibrotic ingredient (Wu et al., 2010). Therefore, we screened out the component in zedoary turmeric oil that affected the activity of HSC in TGF-β1-induced LX-2 (Figure 1A). The western blot results showed that both GER and curdione (CUR) had more obvious inhibitory effect on the expression of collagen I than the other components. And the curcumol was reported to have anti-fibrotic effect (Jia et al., 2018). Furthermore, we examined the effects of GER and CUR on the viability of L02 and LX-2 cells. The results showed that CUR (10–200 μM) did not affect the viability of L02 and LX-2 cells (Figures 1B,C). Although GER (10–200 μM) did not affect the viability of L02 cells, higher concentrations of GER (60–120 μM) inhibited the viability of LX-2 cells (Figures 1D,E); therefore, we continued to study the related effects of GER on LX-2 cells.

### GER Inhibits the Activation of HSCs *in vitro* by Targeting the TGF-β/Smad Signaling Pathway

**FIGURE 2:**
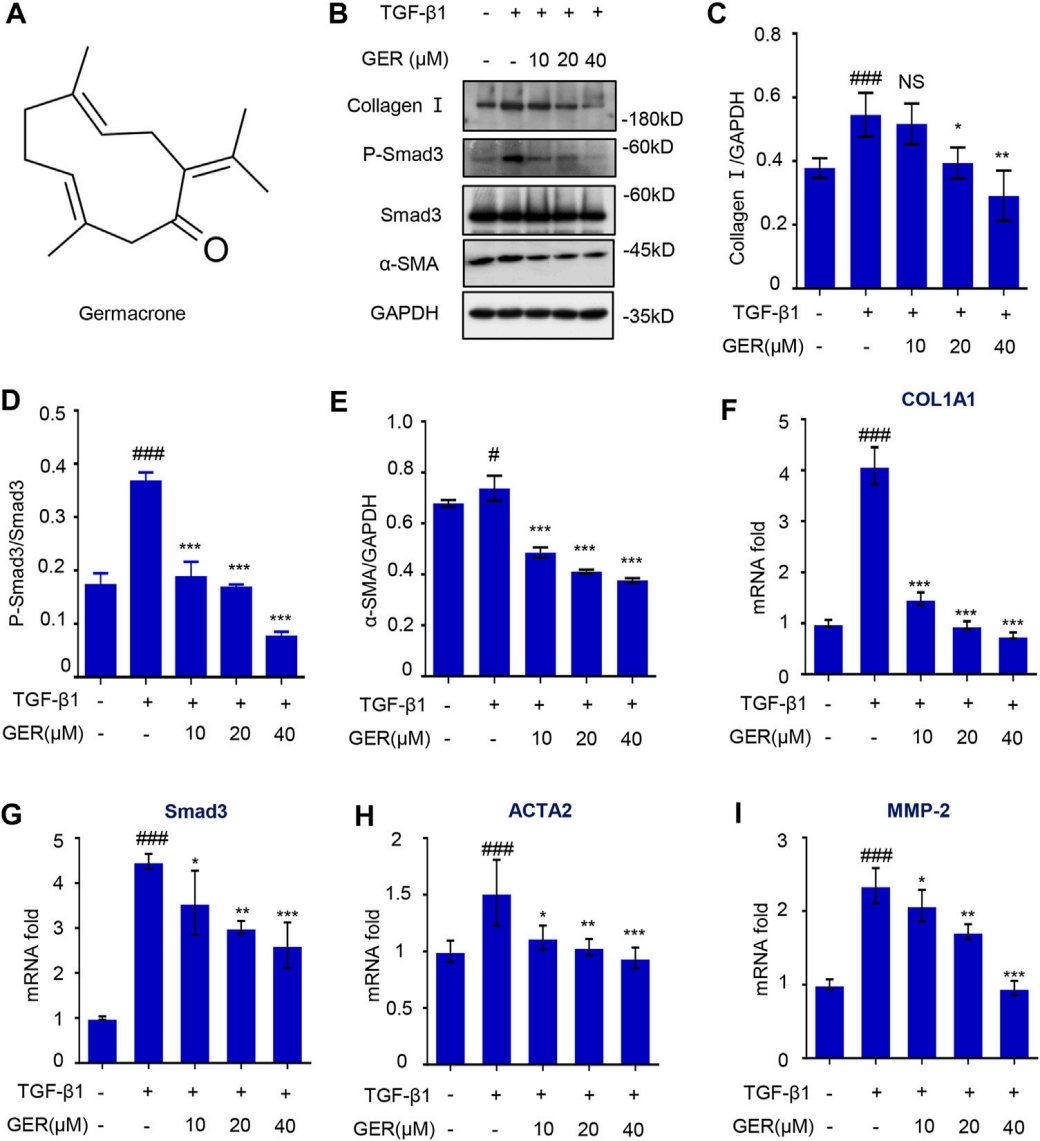
GER suppresses TGF-β induced HSCs activation by inhibiting TGF-β/Smad signaling pathway. **(A)** The molecular structure of GER. **(B)** Western blot for collagen I, P-Smad3, Smad3, α-SMA protein level in LX-2, and GAPDH was used to as the loading control. **(C–E)** Quantitative analysis of collagen I, P-Smad3, Smad3, α-SMA, and GAPDH expression (*n* = 3). **(F-I)** Quantitative real-time PCR analysis of COL1A1, Smad3, ACTA2, and MMP-2 mRNA expression in LX-2. The _ΔΔ_C_t_ method was used to quantify relative changes (*n* = 3). All data are presented as means ± SEM. ^#^ *p* < 0.05, ^###^ *p* < 0.001 vs. the control group. ^*^ *p* < 0.05, **p < 0.01, ***p < 0.001 vs. the TGF-β group. NS, non-significant vs. the TGF-β group.

To explore whether GER could inhibit the activation of HSCs by blocking the TGF-β/Smad signaling pathway, we conducted related experiments on LX-2 cells. First, the effect of GER on the viability of LX-2 cells was measured; the results showed that the concentration of GER below 60 μM had no cytotoxic effects on LX-2. To avoid the influence of cytotoxicity on the experimental results, the concentration of GER below 60 μM was selected for *in vitro* experimental research. Western blot analysis showed that GER reduced the TGF-β1–induced protein level of p-Smad3 and Smad3 in a dose-dependent manner, which directly regulating the expression of α-SMA and collagen I proteins in LX-2 (Figures 2B–E). furthermore, The RT-qPCR analysis revealed that GER significantly decreased the mRNA level of Smad3 and also reduced the mRNA level of its downstream genes ACTA2, MMP-2, and COL1A1 (Figures 2F–I). These results indicated that GER can inhibit the activation of HSCs and the expression of ECMs through the TGF-β/Smad3 signaling pathway.

### GER Inhibits the Survival of HSCs by Regulating Apoptotic Pathway *in vitro*

**FIGURE 3:**
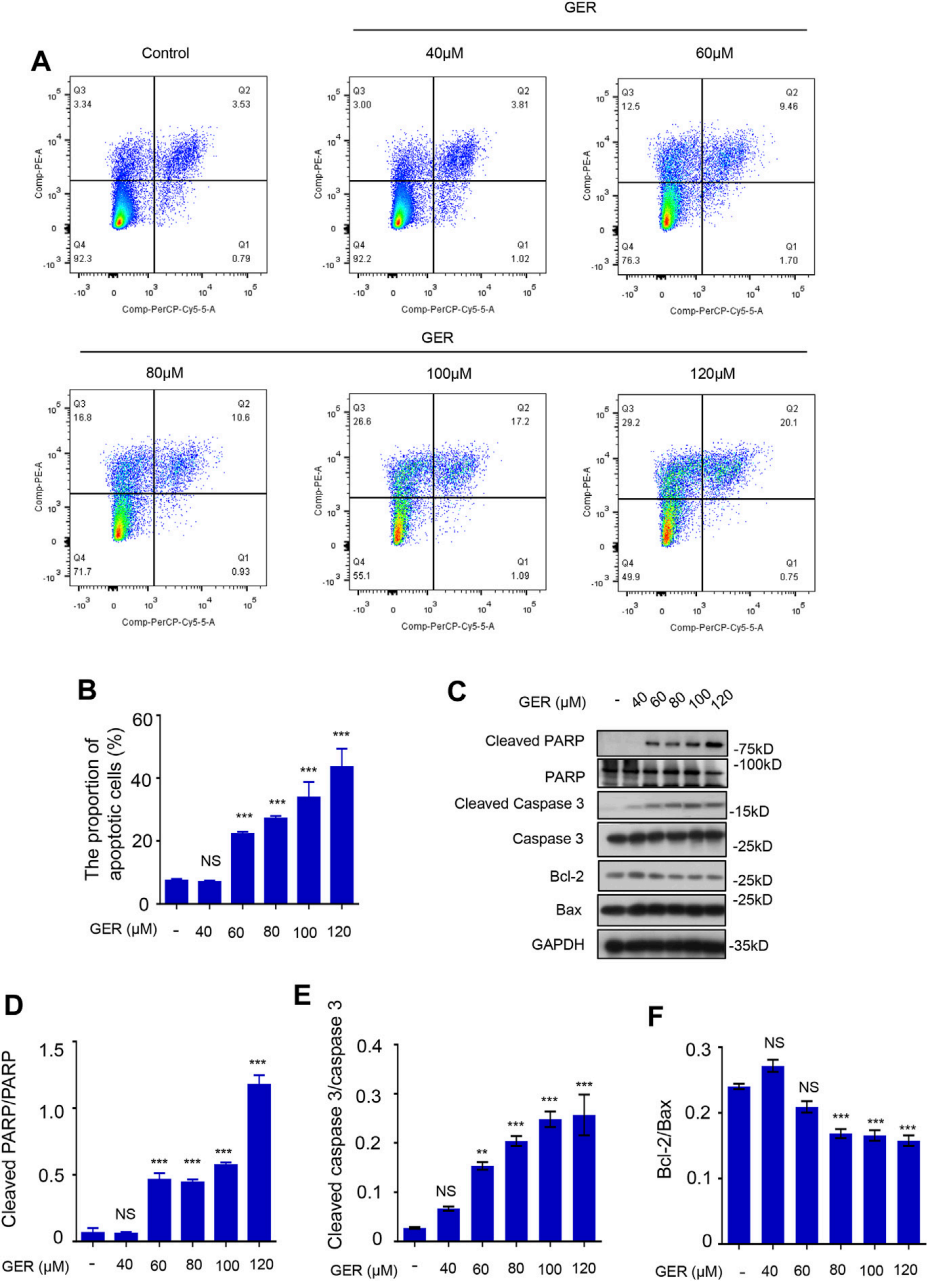
GER promotes the apoptosis of HSCs. **(A)** Flow cytometric analysis of PI-stained apoptotic cells, and the cells in the upper right quadrant (Q2) were early apoptotic cells, and the cells in the upper left quadrant (Q3) were mid and late apoptotic cells. The apoptotic cells contained early, mid and late apoptotic cells. **(B)** Quantitative analysis of different group apoptotic cells after treatment with GER (*n* = 3). **(C)** Western blot for cleaved PARP, PARP, cleaved Caspase-3, Caspase-3, Bcl-2, Bax protein level in LX-2, and GAPDH was used as the loading control. **(D–F)** Quantitative analysis of cleaved PARP vs PARP, cleaved Caspase-3 vs. Caspase-3 and Bcl-2 vs Bax expression (*n* = 3). All data are presented as means ± SEM. ^*^ *p* < 0.05, **p < 0.01, ***p < 0.001 vs. the control group. NS, non-significant vs. the control group.

Previously, we observed that GER (10–40 μM) inhibited the activation of LX-2 cells; however, when the concentration of GER increased, it significantly inhibited the viability of LX-2 cells. Therefore, we explored the role of GER (40–120 μM) in apoptosis of LX-2 cells. First, flow cytometry was used to detect and analyze the proportion of apoptotic cells after GER treatment. The results showed that the proportion of early apoptotic cells (in the upper right quadrant, Q2) and the mid and late apoptotic cells (in the upper left quadrant, Q3) in the GER group were increased in a dose-dependent manner compared with those in the control group, indicating that apoptosis of LX-2 was promoted only by higher concentrations of GER (60–120 μM) (Figures 3A,B). Furthermore, we detected the expression of relevant apoptotic proteins in LX-2 cells. Poly ADP-ribose polymerase (PARP), a DNA repair enzyme and the main cleavage substrate of caspase-3, is the core member of the apoptosis pathway; it plays an important role in DNA damage repair. Namely, the PARP substrate is cleaved by caspase-3 and becomes a form of cleaved PARP that loses its enzymatic activity (Morris et al., 2018). The increased cleaved PARP accelerates cell apoptosis. At the same time, Bax/Bcl-2 ratio is also important. Bax is a proapoptotic protein, which is antagonistic to Bcl-2 (Moldoveanu et al., 2020). In our study, western blot analysis was used to detect the expression of apoptotic proteins; we showed that GER (60, 80, 100, 120 μM) promoted the expression of cleaved PARP and cleaved caspase-3 proteins, and inhibited the expression of antiapoptotic protein Bcl-2 (Figures 3C–F). These results revealed that GER inhibited the survival of HSCs by regulating the apoptotic pathway in a dose-dependent manner.

### GER Protects Hepatocytes by Inhibiting the Production of Mitochondrial ROS *in vitro*

**FIGURE 4:**
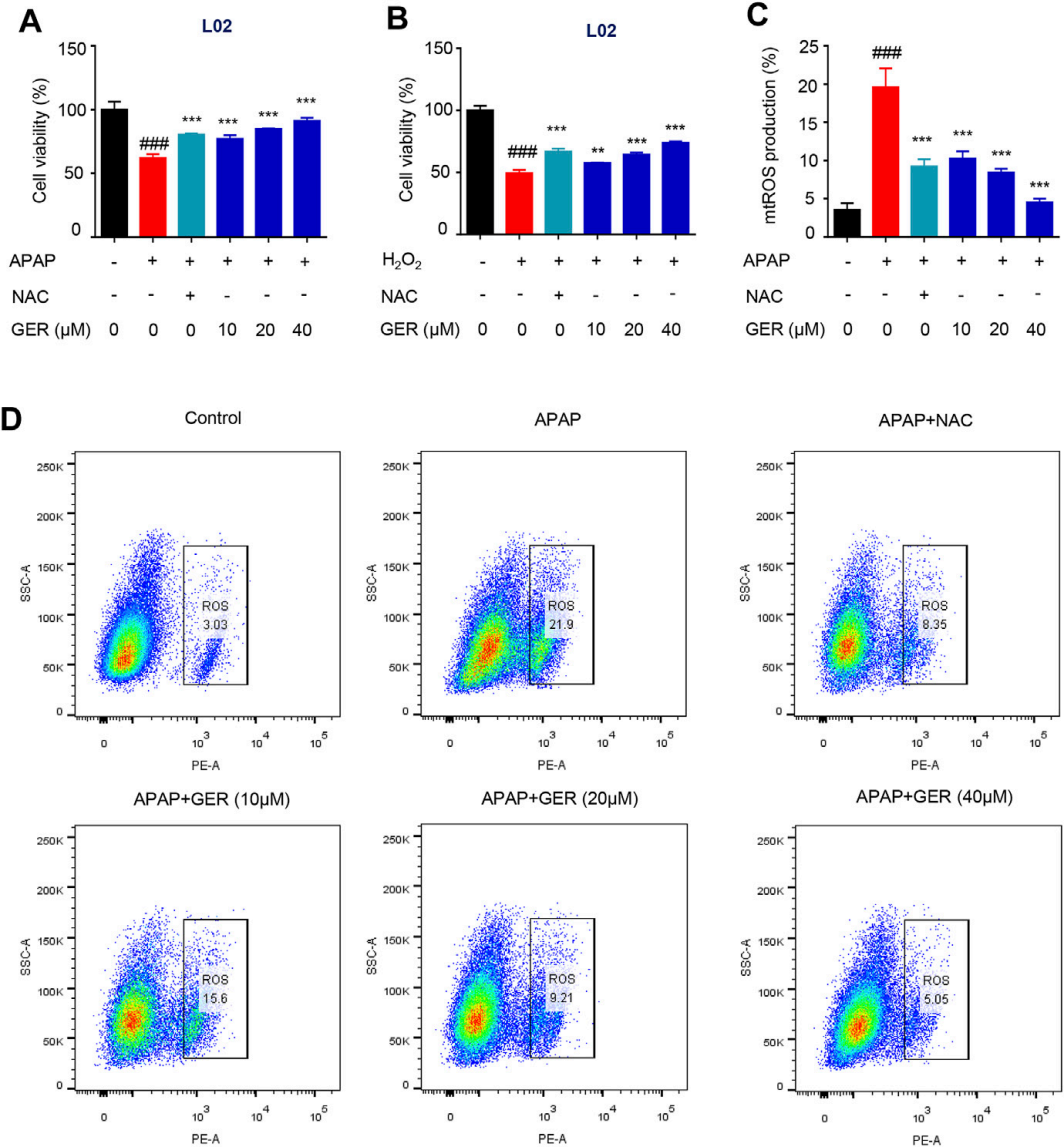
GER protects the hepatocyte by inhibiting the release of mitochondrial ROS *in vitro*. **(A)** L02 cells were treated with APAP (20 mM) for 12 h, and then were treated with GER or NAC for 24 h, then the cell viability was detected by CCK-8 (*n* = 3). **(B)** L02 cells were treated with H_2_O_2_ (600 μM) for 12 h, and then were treated with GER or NAC for 24 h, then the cell viability was detected by CCK-8 (*n* = 3). **(C)** Quantitative analysis of different group mitochondrial ROS release (*n* = 3). **(D)** Flow cytometric analysis of the cell proportion of mitochondrial ROS in L02. The NAC was used as positive control, every group was treated with APAP except for control group. All data are presented as means ± SEM. ^###^ *p* < 0.001 vs. the control group. ^**^ *p* < 0.01, ^***^ *p* < 0.001 vs. the APAP group.

It has been reported that GER may protect hepatocytes (Hashem et al., 2021). In our study, we explored the hepatoprotective effects of GER in APAP cellular damage model. According to the results of preliminary experiments, the cells were treated with 20 mM APAP or 600 μM H_2_O_2_ to induce the hepatocyte damage model and then incubated with different concentrations (10, 20, and 40 μM) of GER. Cell viability was detected 24 h later. The results showed that GER increased the cell survival rate of LO2 cells after APAP or H_2_O_2_ treatment in a dose-dependent manner, confirming the protective effect of GER on hepatocytes (Figures 4A,B). APAP mainly causes mitochondrial oxidative stress to produce ROS, which leads to mitochondrial damage and thus induces liver cell apoptosis. Therefore, we explored whether GER can inhibit the generation of ROS caused by APAP and protect hepatocytes. The MitoSOX red mitochondrial superoxide indicator (Ex/Em: 510/580 nm) was used to stain the cell mitochondrial ROS, followed by detection and analysis through flow cytometry. The flow cytometric results showed that APAP promoted the production of mitochondrial ROS, and the administration of GER was able to inhibit the production of mitochondrial ROS (Figures 4C,D), demonstrating that GER protected hepatocytes from damage by inhibiting the production of mitochondrial ROS.

### GER Ameliorates MCD Diet-Induced Liver Function and Tissue Damage

**FIGURE 5:**
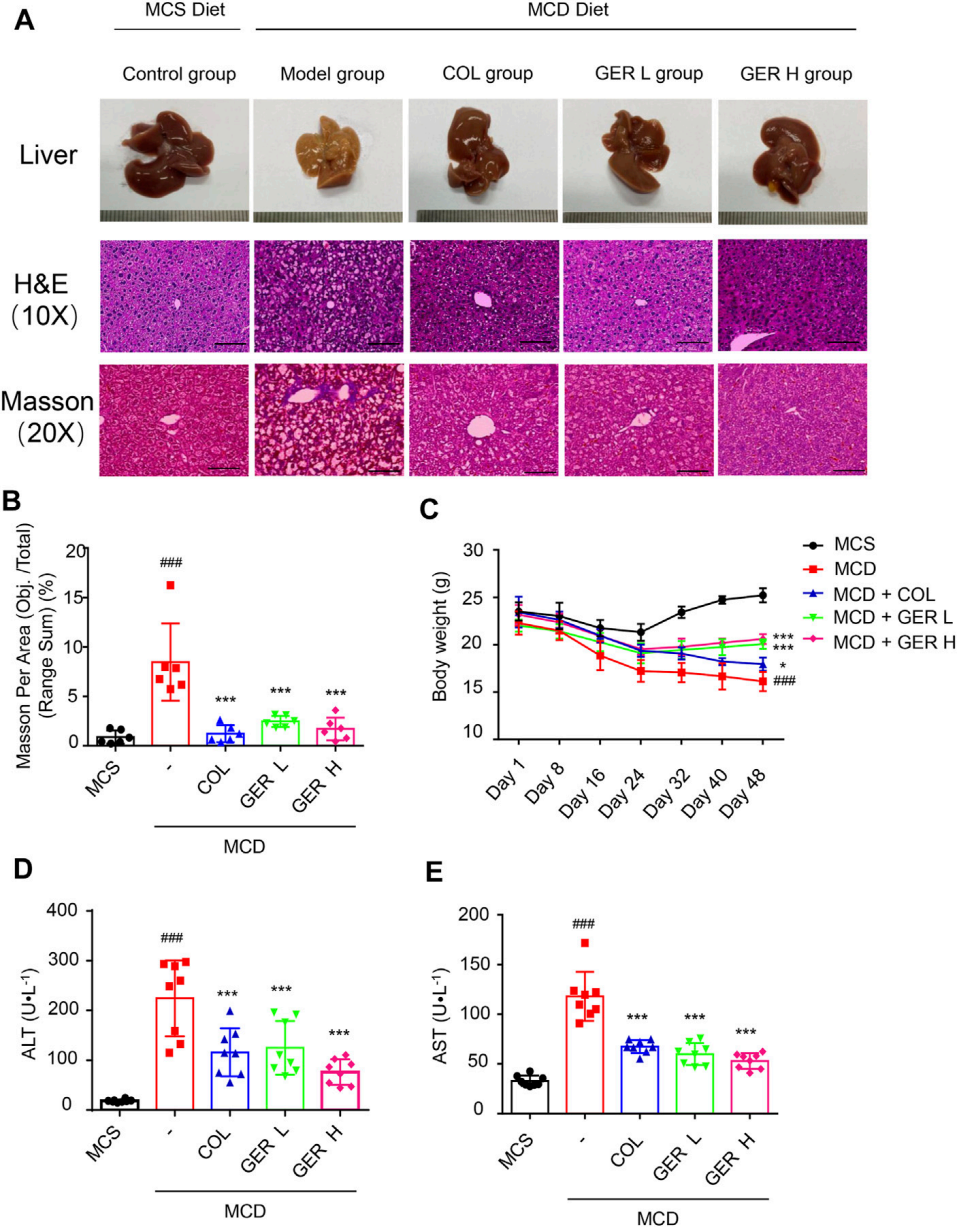
GER ameliorates MCD diet-induced liver tissue and function damage. **(A)** Representative images of the liver and representative images of the liver sections stained with haematoxylin and eosin (H&E, scale bars, 50 μm) and masson (scale bars, 100 μm). Colchicine (COL) was used as positive control, GER L: the low dose (25 mg/kg) of GER group, GER H: the high dose (50 mg/kg) of GER group. **(B)** Quantification of masson changes in different treatment groups (*n* = 6). **(C)** The weight changes of mice in different group for per 7 days **(D,E)** Serum alanine transaminase (ALT) and aspartate transaminase (AST) levels (*n* = 8). All data are presented as means ± SEM. ^###^ *p* < 0.001 vs. the MCS group. ***p < 0.001 vs. the model group.

To explore the role of GER in the process of liver fibrosis, C57BL/6 mice were fed the MCD diet to induce liver fibrosis, and the MCS diet was used for comparison. As shown in Figure 5, compared with the MCS diet group, the liver surface in the MCD model group was rougher, with many nodular textures, and yellowish in color, which situations in GER treatment group were improved effectively (Figure 5A). Similarly, histopathological staining (H&E and Masson) revealed that the liver sections of the MCD diet model group were severely damaged, exhibiting vesicular steatosis, ballooning degeneration of hepatocytes, and fibrosis around hepatocytes and sinuses in the liver; in contrast, the GER group had less liver damage, less steatosis, and less fibrosis (Figures 5A,B). In addition, the weight of the mice on the MCD diet, especially in the MCD model group, showed a downward trend compared with the MCS diet group. After treatment with GER in MCD diet, the weight first showed a tendency to decrease and then to increase, demonstrating that the body function of the mice in the MCD diet group was restored after the administration of GER (Figure 5C). The serum levels of ALT and AST in the MCD model group were significantly higher than those in the control group; in contrast, the levels of ALT and AST were significantly reduced after GER administration, indicating that GER ameliorated liver damage caused by the MCD diet (Figures 5D,E).

### GER Inhibits the Activation of HSCs by Regulating TGF-β/Smad Signaling in MCD Diet-Induced Liver Fibrosis Model

**FIGURE 6:**
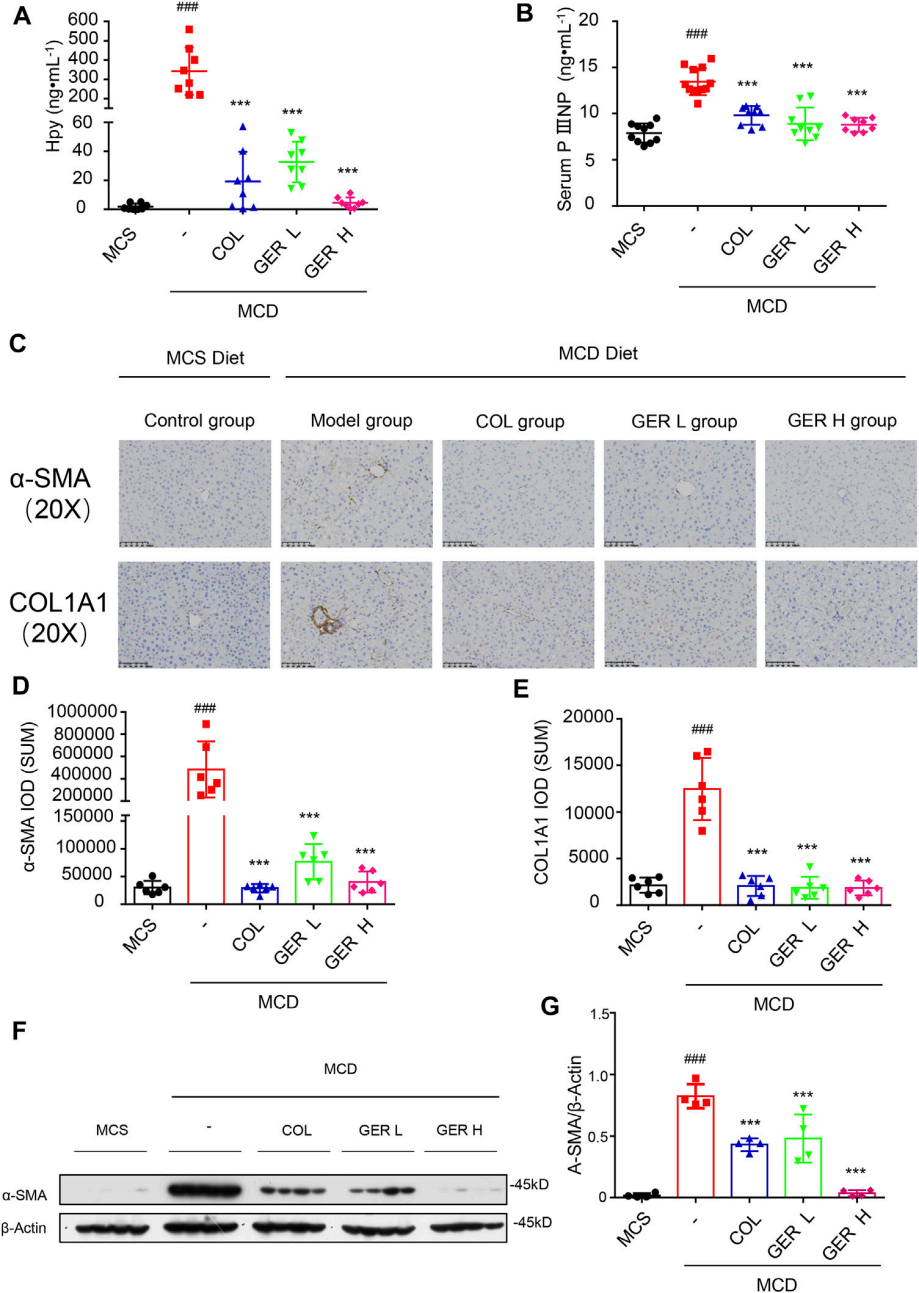
GER inhibits the activation of HSCs via TGF-β/Smad signaling pathway *in* MCD diet-induced liver fibrosis model. **(A)** The content of hydroxyproline in liver tissue (*n* = 8). **(B)** The serum content of PⅢNP (*n* = 8−11). **(C)** Immunohistochemistry for α-SMA and COL1A1 in liver sections (scale bars, 100 μm). **(D–E)** Quantification of α-SMA and COL1A1 expression in different treatment groups (*n* = 6). **(F)** Western blot for α-SMA and Smad3 protein levels in liver tissue, and β-actin was used as the loading control (*n* = 4). **(G)** Quantitative analysis of α-SMA and β-actin expression (*n* = 4). All data are presented as means ± SEM. ^###^p < 0.001 vs. the MCS group. ^*^p < 0.05, ***p < 0.001 vs. the model group.

Hydroxyproline is a characteristic biochemical marker that can be used to quantify the content of collagen in tissues, which is often used to indicate the degree of liver fibrosis (Lee et al., 2005; Gjaltema and Bank, 2017). PIIINP is a cleavage product of procollagen III, which can be detected in serum and used as a circulating biomarker of ECM remodeling in the process of liver fibrosis (Mosca et al., 2019). Therefore, we detected the content of hydroxyproline in the tissue and PIIINP in serum. The results showed that the levels of hydroxyproline and PIIINP in the MCD model group were higher than those in the MCS group, whereas the concentration of hydroxyproline in the tissue and PIIINP in serum were significantly decreased in the GER group, indicating that GER had an antifibrotic effect (Figure 6A,B).

**FIGURE 7:**
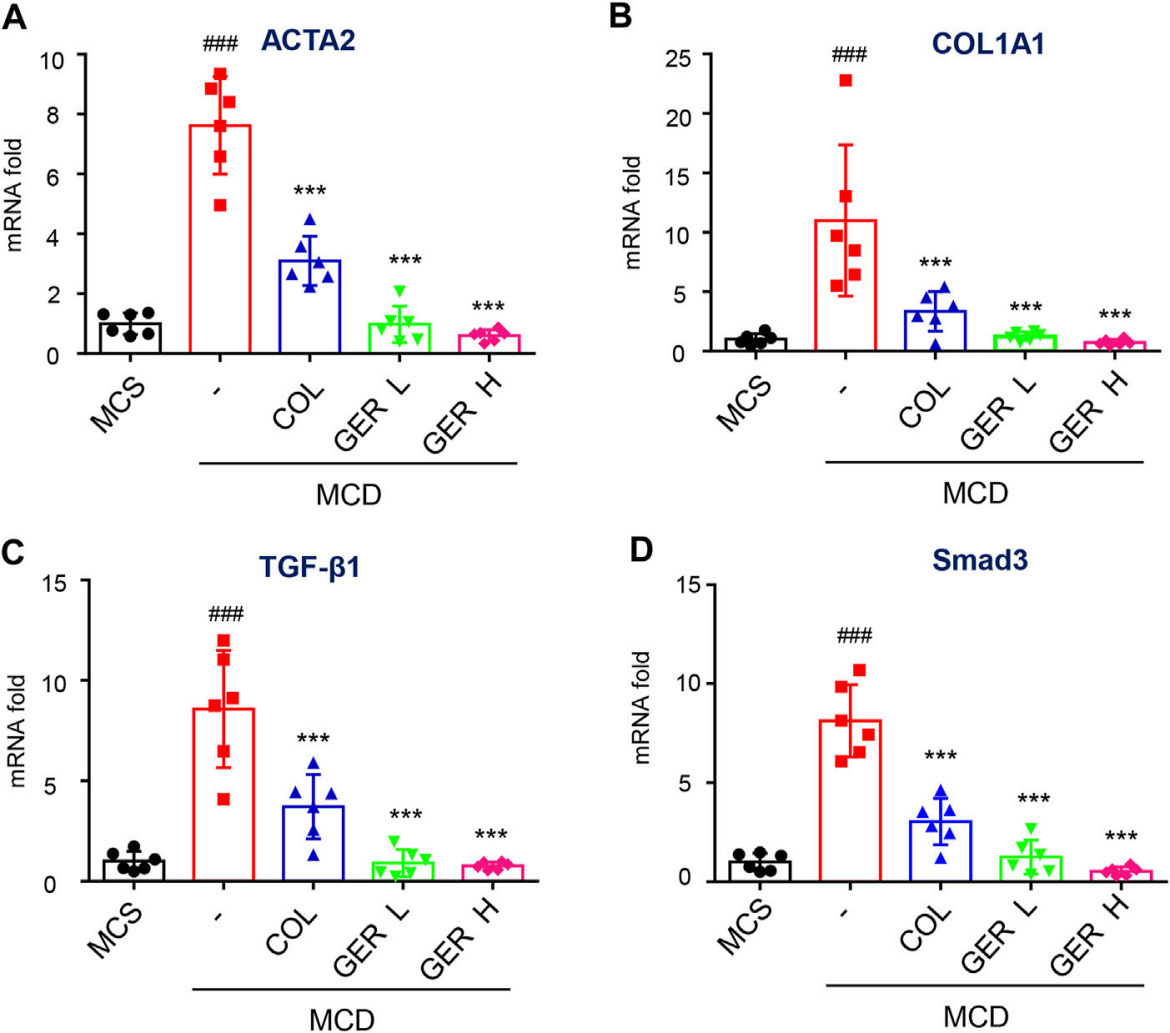
GER inhibits the activation of HSCs via TGF-β/Smad signaling pathway *in* MCD diet-induced liver fibrosis model. **(A–D)** Quantitative real-time PCR analysis of ACTA2, COL1A1, TGF-β, and Smad3 mRNA expression in liver tissue (*n* = 6). All data are presented as means ± SEM. ^###^ *p* < 0.001 vs. the MCS group. ^*^p < 0.05, ***p < 0.001 vs. the model group.

To further clarify the role of GER in the MCD diet-induced liver fibrosis model, immunohistochemical staining was used to analyze the expression of α-SMA and COL1A1 in liver tissues. We found a large amount of COL1A1 accumulation in the liver tissue of the MCD model group compared with the MCS group, while the distribution of COL1A1 was significantly reduced in the GER group, indicating that GER was able to reduce the expression of COL1A1 in liver fibrosis and reduce the deposition of collagen in fibrotic tissue (Figures 6C–E **)**. We also detected the distribution of α-SMA, a marker of HSC activation, in the tissues. The results showed that compared with the MCD model group, the distribution of α-SMA in the liver tissues was reduced after the administration of GER (Figures 6F–G), demonstrating that GER was able to inhibit the expression of α-SMA and block HSC activation in mice with liver fibrosis. Furthermore, we extracted RNA from liver tissue and detected the expression of COL1A1 and ACTA2 genes by RT-qPCR. Consistent with the immunohistochemical results, the expression levels of COL1A1 and ACTA2 in the mice liver tissues of the GER group were lower than those of the MCD model group (Figures 7A,B). Therefore, it was evident that GER inhibited the activation of HSCs in MCD diet-induced fibrosis, reduced the expression of COL1A1, and thereby improved liver fibrosis.

The TGF-β/Smad signaling pathway activation is the main pathway for HSC activation and ECM generation. To explore the cause of liver fibrosis in the MCD diet model and the mechanism of GER against liver fibrosis, we determined the content of TGF-β in serum and tissues. The results of RT-qPCR showed that the TGF-β levels in liver tissue in the MCD model group were significantly higher than those in the MCS group and the GER group (Figure 7C), indicating that the increase in the TGF-β level was the main cause of liver fibrosis induced by MCD diet. In addition, the GER group significantly reduced Smad3 protein expression compared with the MCD model group (Figure 7D). The above results demonstrated that GER was able to reduce the increase in TGF-β levels caused by MCD diet and to suppress the activation of HSCs through the TGF-β/Smad signaling pathway.

## Discussion

Fibrosis is a part of the wound healing response after catastrophic tissue damage, which maintains the integrity of organs, but can also lead to a variety of human pathological conditions, including cirrhosis (Bataller and Brenner, 2005; Lee et al., 2015). The reversal and treatment of liver fibrosis is an important means to reduce the severity of chronic liver disease; therefore, there is an urgent clinical need to develop an effective antifibrotic drug (Iredale, 2007; Roehlen et al., 2020). GER, which was screened out from zedoary turmeric oil in this study, is a kind of open double-ring sesquiterpene compound with ketone group in turmeric plants (An and Jang, 2020); it has numerous physiological activities and great medicinal potential, including anti-inflammatory, anti-virus, anti-tumor, and anti-oxidant effects (Aggarwal et al., 2013; An and Jang, 2020). In the last years, the mechanisms of antifibrotic effect were investigated. The results showed that the activation of HSCs into proliferative fibro/myo-fibroblasts has been found to be the central factor of liver fibrosis, and they found that the expression of α-SMA, a HSCs activation marker, and collagen I were increased after stimulating by TGF-β1 (Dewidar et al., 2019). However, few studies reported the effect of GER on liver fibrosis. In this study, GER inhibited the expression of α-SMA in TGF-β1-stimulated LX-2, indicating that the activation of HSCs might be prevented by GER.

TGF-β1, the most powerful fibrocytokine in the microenvironment of liver fibrosis (Hellerbrand et al., 1999), exerts its biological activity via the Smad signaling pathway. Smad signal transduction pathways mediated TGF-β1-induced collagen synthesis through the phosphorylating Smad2/3, which can translocate to the nucleus to regulate the target genes, and thus play a crucial role in the development of liver fibrosis (Roberts et al., 2006). To explore whether GER regulates liver fibrosis through TGF-β/Smad signaling pathway, the GER attenuated TGF-β1-induced LX-2 fibrosis was used. Ours results showed that both the protein level of phosphorylating Smad3 and Smad3 were decreased after GER treatment. Furthermore, the mRNA level of Smad3 and its downstream gene ACTA2, COL1A1, and MMP-2 were also downregulated. These results indicated that inhibiting of TGF-β/Smad signaling pathway may be a key mechanism to GER exerts its anti-fibrotic effect in TGF-β1-induced LX-2.

Inducing apoptosis of HSCs and inhibiting the survival of HSCs are also important mechanisms of anti-hepatic fibrosis. Various growth factors released by damaged hepatocytes are the main reasons for the rapid proliferation and activation of HSCs. On the one hand, the proliferation of HSCs is positively regulated by PDGF, on the other hand, it inhibits its apoptosis (Tsuchida and Friedman, 2017). Therefore, we can also inhibit the survival of HSCs by promoting the apoptosis of HSCs. It was reported that Gliotoxin promotes the apoptosis of HSCs by activating Caspase 3 and consuming ATP, thus improving liver fibrosis in rats (Dekel et al., 2003). Ours results showed that the dose of GER plays a regulatory effect on HSCs. At the low dose (lower than 40 μM), GER can inhibit the activation of HSCs through the TGF-β/Smad signaling pathway. However, when the concentration of GER is in the range of 60–120 μM, it can promote the apoptosis of HSCs by upregulating Cleaved-caspase 3 protein expression and inhibiting anti-apoptotic protein Bcl-2 expression as well as has no toxic effect on hepatocytes. These indicates that GER has a dual effect on HSCs, inhibiting the activity of HSCs in the therapeutic concentration range to exert an antifibrotic effect. When the concentration of GER increases, it can induce programmed apoptosis of HSCs, which can effectively inhibit the survival of HSCs in the process of fibrosis.

Liver injury contributes to liver fibrosis where hepatocytes change their gene expression and secretion profile in response to such injury, and the newly expressed fibrogenic factors including TGF-β and NADPH oxidase 4 (Lan et al., 2015; Wang et al., 2016). Nox4 mediates the synthesis of ROS that is also one of the reasons for the activation of HSCs (Novo et al., 2011). A large number of studies have shown that most of liver fibrosis *in vivo*, including CCl_4_, alcohol, Thioacetamide (TAA) and nonalcoholic steatohepatitis (NASH), injured hepatocytes proposed to play a causative role in the induction of liver fibrosis (Ramos-Tovar and Muriel, 2020). Furthermore, it was found that HSCs cultured with ROS produced by stimulated neutrophils showed that an increased level of procollagen mRNA and protein (Casini et al., 1997). Therefore, ROS is also the one of the accomplices in liver fibrosis. In this study, APAP was used to produce ROS in L02, and we found that GER can protect hepatocytes and reduce the release of ROS from injured hepatocytes *in vitro*. And *in vivo* experiment showed that GER significantly improved the state of liver injury in mice with lower serum aminotransferase subjected level to MCD-diet group; through observing the pathological sections, we found that the hepatocytes with fatty degeneration and fat vacuoles of steatosis in the GER group were significantly less common. All these findings indicate that GER may not only directly prevent the damage of parenchymal cells, but also inhibit the activity of stellate cells by inhibiting the release of damage substances and ROS to attenuate liver fibrosis.

So according to *in vitro* study, We found that the effect of GER on attenuating liver fibrosis can be explained from two aspects. On the one hand, its direct effect is to inhibit the activation and survival of HSCs. In terms of what they have in common, they all act directly on HSCs to inhibit liver fibrosis, but the difference is that the concentration of GER effect is different, and the mechanism is also different. On the other hand, GER can indirectly prevent liver fibrosis by protecting hepatocytes. At the same time, the two effects of GER inducing HSCs apoptosis and GER protecting hepatocytes are synergistic in preventing liver fibrosis.

MCD-diet mice is a well-established nutritional model of liver fibrosis with serum aminotransferase elevation, and liver histological changes similar to human Non-alcoholic steatohepatitis (Li et al., 2018). To explore the effect of GER on hepatic fibrosis, a MCD-diet model of liver fibrosis was investigated. According to related research, MCD-diet can lead to abnormal liver metabolism, massive fat accumulation and weight loss (Li et al., 2020). But in our study, the mice weight showed that GER could prevent MCD-diet induced weight loss, and we detected lower level of ALT and AST in GER group, in addition, histopathological analysis showed that there was less vesicular steatosis, ballooning degeneration of hepatocytes, and fibrosis around hepatocytes and sinuses in the liver of the GER group. Consistent with *in vitro* data, GER could protect liver through reducing the release of ROS produced by damaged hepatocytes. These results indicated that GER may reduce liver injury, balance the level of oxidative stress in the liver, and then improve the liver function to play the anti-fibrotic effect.

Levels of hydroxyproline and PIIINP in liver are the important indicators reflecting the degree of liver fibrosis. In our study, MCD-diet mice showed increased levels of hydroxyproline and PIIINP in liver, which were significantly decreased after GER administration. Therefore, we further explored the anti-fibrotic mechanism of GER *in vivo*. Immunohistochemical analysis showed that there were a large number of α-SMA and COL1A1 proteins distributed in MCD-diet mice liver, but both of them were decreased after GER treatment. This phenomenon was consistent with the results of our *in vitro* experiments, which further confirmed that GER inhibited the activity of HSCs. RT-qPCR analysis showed that the gene level of TGF-β was upregulated in MCD-diet mice liver, at the same time, its transduction signal pathway Smad 3 and its downstream genes ACTA2 and COL1A1 were also upregulated, which indicating that TGF-β/Smad signaling pathway was a important mechanism in MCD-diet induced liver fibrosis. However, GER could reduce the expression of TGF-β, Smad 3, ACTA2 ,and COL1A1 genes, so that inhibited TGF-β/Smad signaling pathway. The same results had been confirmed on LX-2.

In conclusion, we found GER could attenuate liver fibrosis through regulating multiple signaling pathways, the first was to inhibit the activity of HSCs through TGF-β/Smad signaling pathway, the second was to protect the liver, reduce hepatocytes injury and liver oxidative stress, and avoid ROS-induced the activation of HSCs. Finally, the survival of HSCs was inhibited through apoptosis pathway. To a certain extent, GER may be used in the treatment of clinical chronic hepatic fibrosis.

## Data Availability

The original contributions presented in the study are included in the article/supplementary files, further inquiries can be directed to the corresponding authors.
